# Supplementary material for: Bacteriophage-mediated reduction of uropathogenic E. coli from the urogenital epithelium
Source: Infect Immun. 2026 Feb 26;94(4):e00543-25. doi: 10.1128/iai.00543-25 (PMC13081728; doi:10.1128/iai.00543-25)
Supplement: Supplemental material — Fig. S1 and S2; Supplemental movie captions. [file iai.00543-25-s0001.pdf]

SUPPLEMENTAL MATERIALS

Bacteriophage-mediated reduction of uropathogenic *E. coli* from the urogenital epithelium

Bishnu Joshi, Jacob J. Zulk, Camille Serchejian, Zainab A. Hameed, Addison B. Larson, Austen L. Terwilliger, Deepak Kumar, Indira U. Mysorekar, Robert A. Britton, Anthony W. Maresso, Kathryn A. Patras

Contents:

Supplemental Figures 1-2
Captions for Supplemental Videos 1-2

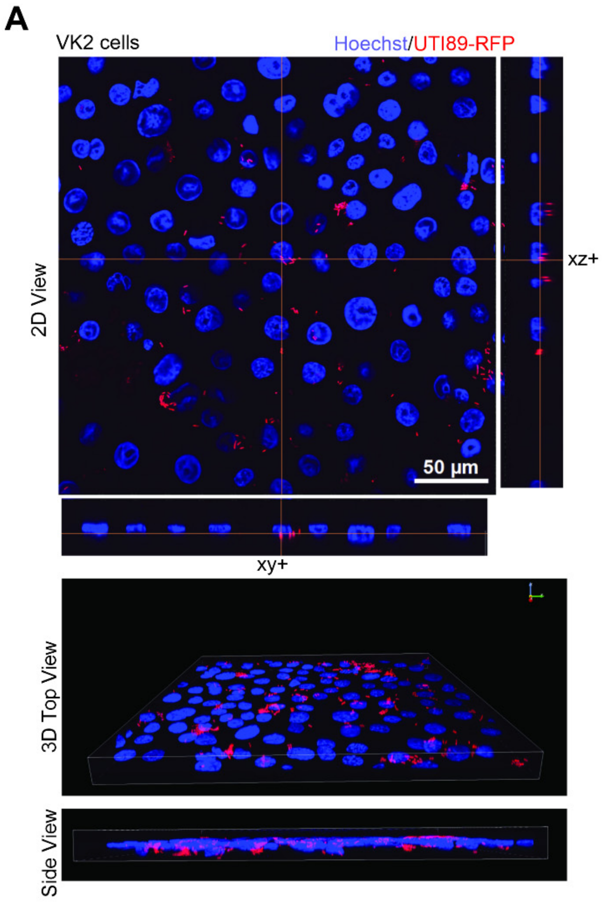

Supplemental Figure 1. Orthogonal projections of z-stack images showing intracellular UPEC in VK2 and HTB-9 cells. VK2 and HTB-9 cell monolayers were infected with UPEC strain UTI89-RFP (red) and incubated for 3 h. Three-dimensional orthogonal projections of the z-stack are shown in two different planes to visualize intracellular bacteria within the cell layers. Nuclei were stained with Hoechst (blue). Scale bar = 50 µm.

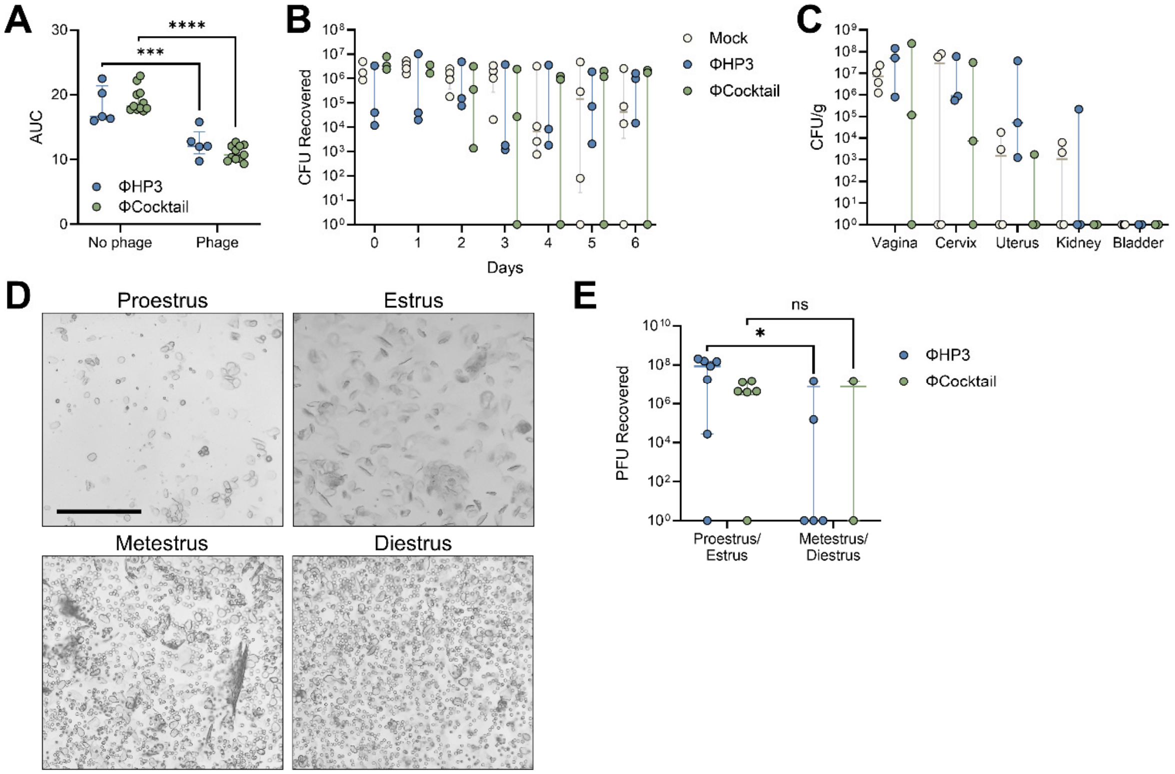

Supplemental Figure 2. Phage susceptibility of UPEC colonies recovered post-treatment and impact of lower UPEC inoculum and estrous stage on outcomes. (**A**) UPEC colonies were isolated from vaginal swabs collected after 4-6 days post-phage treatment. Six randomly selected colonies were cultured overnight in LB broth, diluted, and subsequently infected with ΦHP3 or ΦCocktail at an MOI of 10, or no phage as a control. Growth was monitored by measuring OD_{600nm} at 15 min intervals for 20 h, and area under the curve (AUC) values were calculated. One day after estrous cycle synchronization, ^{HMb}mice were intravaginally inoculated with UTI89 $Strep^R$ (10^5 CFU) and 24 h later, mice received an intravaginal dose of either 10^8 PFU of ΦHP3 or ΦCocktail, or phage buffer only as a control. (**B**) UTI89 $Strep^R$ CFU recovered from vaginal swabs over daily treatments. (**C**) UTI89 $Strep^R$ CFU/g of urinary and reproductive tissues at day 7 post-phage treatment. (**D**) Representative images of estrous staging of mouse vaginal lavage fluid showing dominant cell types in proestrus (nucleated epithelial cells), estrus (Anucleated keratinized epithelial cells), metestrus (all three cell types), and diestrus (neutrophils). Images were collected at 100X, scale bar = 200 µm. (**E**) After estrous staging, mice were vaginally inoculated with 10^8 PFU of ΦHP3 or ΦCocktail. PFU was quantified from vaginal swabs 4 h post-treatment and mice were grouped by estrous stage at the time of phage inoculation. Points represent means of individual colony replicates (A) or individual mice (B, C, E), and lines represent medians with interquartile ranges. Experiments were performed once in technical duplicate (A) or with n=3-4 (B-C) and n=2-7 (E). Data were analyzed by two-way ANOVA with Šídák's multiple comparisons test (A, E) or mixed-effects model with Dunnett's multiple comparisons test (B-C), * p<0.05, ***p<0.001, ****p<0.0001.

Supplemental Video 1. ΦHP3 phage adherence to human vaginal epithelial VK2 cells, related to Figure 2. SYBR-Gold–labeled ΦHP3 (green, arrow) were applied to VK2 cell monolayers, and time-lapse images were acquired immediately after phage addition at 30 s intervals for a total of 10 min. Cells were counterstained with Hoechst (nuclei, blue) and wheat germ agglutinin (membrane, magenta), scale bar = 10 μm.

Supplemental Video 2. ΦHP3 phage adherence to human bladder carcinoma HTB-9 cells, related to Figure 3. SYBR-Gold–labeled ΦHP3 (green, arrow) were applied to HTB-9 cell monolayers, and time-lapse images were acquired immediately after phage addition at 30 s intervals for a total of 10 min. Cells were counterstained with Hoechst (nuclei, blue) and wheat germ agglutinin (membrane, magenta), scale bar = 10 μm.
